# Puerperal Convulsions

**Published:** 1863-02

**Authors:** 


*Puerperal Convulsions.*—In the proceedings of the Cincinnati Academy of Medicine, reported in the *Lancet & Observer*, we find a discussion on the subject of Puerperal Convulsions, which strikes us, in the main, as scarcely creditable to that enlightened emporium of medical science. The subject was introduced by J. H. Tate, M. D., who read an essay upon it. Dr. T. called attention to the facts that ninety per cent. of the cases occurred in women not broken down by child bearing, or any cachexia; that the disease is almost invariably accompanied by albuminuria; that the eclampsia is very commonly attended by œdema of the legs, hands, and often of the face; that, fourthly, hundreds have been successfully treated by bloodletting and purgatives; fifthly, that choloroform has, recently, in quite a number of cases, proved successful; and finally, that in some cases a rapid emptying of the uterus has been followed by an early cessation of the convulsions. The disclosures of *post mortem* examinations have been unsatisfactory. Hence the writer concludes that bloodletting is the chief remedy, adopting "the views of Meighs and Tyler Smith (?)" The essayist observes:

"In entering upon this task, I have experienced some of the feelings of the lover of nature, who goes forth at twilight to survey the beauties of the night. As gem after gem, and constellation after constellation, rise up in splendor before him, his eye kindles with a deeper and yet more profound emotion, until at length he is overwhelmed and lost amid the grandeur and extent of the universe of God."

Then follows a case by Dr. Carroll, which was bled and died; then a case by Dr. Smith, which was bled and died.

Upon which Dr. Fries recommended bleeding from both arms and delivering by the forceps while the blood was flowing. Dr. Carroll objected to chloroform but endorsed the blood letting. Dr. Richardson believed in chloroform, but had more confidence in bloodletting as a preventive agent, "to remove pressure from the brain." Dr. Murphy had seen three cases, the first was bled and died; the next two were not bled and lived. He (most sensibly) urged the facts of uraemia. Dr. J. B. Smith said:

"Women during pregnancy do not bear a large loss of blood without detriment. There is a physiological condition during menstruation when she loses a certain amount of blood. During pregnancy it is used in the development of the fabric of the child. He took the position and believed it, that unless there was an arrest of development of the child or the uterus, there is no excess of blood. As a remedy for puerperal convulsions, does an excess of blood about the spinal cord indicate bloodletting? His patient died after the last bleeding. Would every drop of blood drawn have relieved her? You must remove the exciting cause. The best authors, as Tyler Smith, Braun and others, are exceedingly cautious about bloodletting, and he would be when œdema was present, indicating albuminuria."

Dr. Fries said nineteen out of twenty women at the seventh, eighth and ninth months will have an œdematous condition of the extremities. This was due, not to the uræmia, but to pressure on the lympathics of the pelvis. Did not believe in poisoning of the blood. Had bled hundreds in such cases with benefit. Pregnant women bear bloodletting well. You could not wait for stimulants or antidotes, but should bleed irrespective of poisons in the blood. "He would open a vein, apply stimulants to the extremities, ice water to the head, etc. In fatal cases, post mortem examinations reveal effusion around the base of the brain or a clot."

Dr. Murphy took grounds against extreme bloodletting and extreme anti-phlogistic treatment. In the Commercal Hospital the treatment of the disease was as successful now, as in the days when bloodletting was practiced largely. Did not believe the doctrine that pregnant women bear bloodletting well. Excessive bloodletting would produce anæmia and convulsions.

Dr. Fries reported a case to which he was called since the commencement of the discussion. He had bled her largely and she died. Would treat a similar case in the same way. Subsequently he inveighed against the New York practice of producing abortion in albuminuria, and said that the theory of albumiuaria or uræmia being the whole cause of puerperal convulsions is a humbug.

Dr. Richardson did not believe there was any one plan of treatment in puerperal convulsions. Favored chloroform, and believed that blood-letting was useful only when there was a rigid, unyielding cervix and os uteri. Bloodletting in advance would be detrimental.

Dr. Murphy agreed with Dr. R. "He was asked how many women he had bled? He said very few. Who bleed now, except such persons as cannot treat typhoid fever without purging and salivation? We cannot bleed these women or give heroic doses of medicine." He could not treat his patients as the older authors taught, simply because they could not bear it. Those who have read the recent investigations on the white kidney will not think so strange of producing abortion in cases of albumiuaria. We have come to know an inflammation is a state of hyper-nutrition.

Dr. Carroll has bled hundreds of cases of typhoid fever. "He bled them because in the outset there is local difficulty in the brain. He had practiced more than forty years, and he never gave stimulants in typhoid fever in the first ten days. If there is congestion of mucous membrane stimulants would increase it. He would rather act quietly on the secretions; and this was the opinion of the best men in the profession. Believed there was not a man in the Edinburgh School who was not a hypocrite. Cullen was the only man, he believed, who kept himself clear. Why must you not bleed in effusion, in œdema! You take a case of dropsy following scarlet fever, when there is danger of convulsions; you will almost always save the patient by bleeding. If you bleed, you relieve the brain. In all cases where the kidneys do not act well, you cup, leech and purge, and you bring on healthy action; by bleeding you lessen the amount of fibrin."

Several of the participants in the discussion spoke with contempt of "reading doctors." It is very clear from their reported remarks, that they have none of that Christian virtue which teaches contempt of self. The "reading doctors" have very small confidence in blood-letting (and very often none at all) in eclampsia. The "non-reading" doctors have bled hundreds of cases, and propose so to continue notwithstanding their patients about invariably die.

But Dr. Carroll caps the climax by saying that not only does he bleed in eclampsia, in fact has bled in hundreds of cases, but that he bleeds in the outset of typhoid fever, "because there is local difficulty of the brain!" *Ohe! jam satis!*

And Dr. Carroll believes that "there was not a man of the Edinburgh school who was not a hypocrite." Evidently Dr. Carroll is not of the despised class of "reading doctors."

It has been well said that a physician should never abandon his care of a patient until he is absolutely dead. And why? Simply because in occasional instances, while the patient seems now in the moribund state, destructive changes will cease a little before those processes which are immediately necessary to the continuance of life. On the cessation of these destructive changes, commonly almost coincident with death, there will sometimes commence recuperative action and convalescence steadily advance. Who has not seen this in cases of, for instance, enteritis, where there has been the usual spasmodic contraction of the intestine? In laryngitis, in meningitis, and many other diseases? A crisis? Certainly a crisis—but one in which Death is the physician. A crisis not to be imitated by art, but to be shrunk from, and avoided by all the appliances of art. This is the kind of crisis which is more dangerous than disease, but which, unfortunately, when one case has happened to escape, has been made the excuse for multitude murders. (For the details of at least one such case, see Paine's "Institutes of Medicine," (Appendix) Pp. 871 and 872, § 1068 d, where the patient was pushed to the death crisis by a blood thirsty dogma, and yet recovered.) Cases involving spasmodic action are especially illustrative of this state of affairs.

This will explain why it is that the Bloodletters always insist, if the patient dies, that he or she was not bled enough. It is true, even as they say—they were not bled enough! If they had been bled until the *death-crisis* came, possibly they might have recovered, but how many will die?

It occurs to the present writer, that there is no warrant in all medical experience, or even in medical theory, for this murderous use of the lancet. Statistics, all experience, the enlightenment of the age, and common sense, stand arrayed upon one side, and upon the other side, cases like Dr. Paine's, (§ 1068 d), which escaped both Death and the Professor, and Dr. Fries', which did die, but illustrated the excellence of the treatment even while so doing.

The simple fact is that blood-letting is a sedative agency, but one rapidly exhaustive in character, and hence liable to objection, in particular cases, which may not weigh against other sedatives. That it is not pre-eminent as a sedative, is proven by the facts of every-day experience. That it is practically inconvenient, unnecessary and liable to result injuriously, is so patent that the lancets of the bloodthirstiest bleeders are everywhere (*nolens volens*) getting rusty from want of use.

In puerperal convulsions there are two prime indications: First, to control the paroxysm. Second, to remove the cause. The cause may be uræmic or reflex, each involving change in the molecular structure of the nervous system. Extravasations, clots, congestions, effusions—these are results, not causes. Pressure is not a cause, it is a result. Bloodletting in these cases, save for its sedative influence, is an absurdity.

Sedatives, anæsthetics, anodynes—these are what fulfill the first indication.

The second may require diruetics, cathartics, or other eliminating agents. It may require tonics, nutriment, alteratives. It may necessitate speedy abortifacients. Nature may and often does provoke this action spontaneously. Art should be her minister and interpreter. It is idle under these circumstances to indulge in canting, milk sop platitudes about the "terrible practice of New York," or any other place, in this regard. It is a question simply of one life or two. It is a question which does not need discussion, save in those countries where it is gravely proposed always to sacrifice the mother rather than the child. It is not a question for this age or this country.

Spoliative blood-letting, however great its claims, when other or less disastrous sedatives were either unknown or not understood, is out of place, save in exceptional instances,—in puerperal convulsions, or, briefly, in any known form of disease.

